# Evidence in Practice of Tissue Healing with Latex Biomembrane: Integrative Review

**DOI:** 10.1155/2019/7457295

**Published:** 2019-03-03

**Authors:** Suélia de Siqueira Rodrigues Fleury Rosa, Mário Fabrício Fleury Rosa, Marcos Augusto Moutinho Fonseca, Glécia Virgolino da Silva Luz, Carlos Federico Domínguez Avila, Aldira Guimarães Duarte Domínguez, Aldene Guimarães Duarte Dantas, Von Braun Richter

**Affiliations:** ^1^Postgraduate Program in Biomedical Engineering-PPGEB at Gama-FGA, University of Brasília-UnB, Brasília 72.444-240, Brazil; ^2^Postgraduate Technology and Health Program, University of Brasília-UnB at Ceilândia-FCE, Brasília 72220-275, Brazil; ^3^Postgraduate Program in Human Rights, Citizenship and Violence/Political Science, University Center Unieuro, Brasília 70.200-001, Brazil; ^4^University of Brasília at Ceilândia-FCE, Brasília 72220-275, Brazil

## Abstract

Wound healing is a perfectly coordinated cascade of cellular, molecular, and biochemical events which interact in tissue reconstitution. Chronic diseases such as pressure ulcers (PU) and diabetes mellitus (DM) are considered risk factors for wound healing. Patients with such diseases often have higher sepsis, infection, and complication rates, since they have revascularization inhibition and low growth factor expression. Thus, latex biomembrane (LBM), a biocompatible material, derived from the latex of the rubber tree (*Hevea brasiliensis*) appears to create tendencies as an angiogenic-inducing tissue healing agent and as biomaterial, resulting from its structural qualities and its low cost when compared to conventional treatments. Therefore, this work aims at summarizing the results, experiments, and scientific findings that certify or recommend the use of LBM as a new technique to be applied effectively in the treatment of wounds. An integrative review was held in the BIREME, LILACS, Burns, MEDLINE, PubMed, and SciELO databases, from 2000 to 2016, using the following descriptors: “healing,” “diabetes mellitus,” “wounds,” and “latex membrane.” As a result, 600 experiments (out of 612) presented satisfactory results; however, 33% of the cases received explicit recommendations, 11% required more studies on the subjects, and 1% was denied. On the other hand, half of the studies did not expressly endorse its use, despite presenting satisfactory results. The LBM was characterized as a good therapeutic alternative in cases of wounds, including chronic diseases, such as diabetes mellitus and PU, due to its relevant potential for wound healing stimulation, acceleration of cell tissue mending and revascularization, or the reestablishment of angiogenic functions (creation of new blood vessels). The LBM was also confirmed to be safe as a biocompatible material whose structural qualities (elasticity, adaptability, impermeability, and possibility of suture), devoid of toxicity, allowed interaction between tissues and presented no hypersensitivity inducer and no antimicrobial effect.

## 1. Introduction

Wound treatment has been evolving since 3,000 BC, when hemorrhagic wounds were treated with cauterization. There are evidences of the use of tourniquets that date back to 400 BC, while suture is from the 3rd century BC. In the Middle Ages, with the creation of gunpowder, wounds became a more serious matter. Nowadays, chronic wounds caused by pressure ulcers (PU), vascular ulcers, and neuropathic ulcers (diabetes mellitus (DM) and leprosy) associated with complications in ambulatory therapeutic efficiency are quite challenging to treat [1–3].

The most common chronic wounds in the American population are chronic ulcers in the lower limbs (80 to 90%), and its etiology is venous stasis, at 5% arterial insufficiency and 2% neuropathy [4]. According to information from the International Diabetes Federation (IDF), 80% of the people with diabetes live in low- and middle-income countries. For example, in South and Central America, there were 26 million patients diagnosed with diabetes in 2017, and it is predicted that this number will increase to 42 million until 2045 [5]. Specifically in Brazil, the amount of patients diagnosed with diabetes has increased 61.8% between the years of 2006 and 2016 [6]. The World Health Organization (WHO) informs that roughly 422 million of adults have diabetes and that 2% of the population of the planet have complications in healing chronic wounds [7].

In Costa et al.'s survey [8] on the cost for PU treatment, treatment material costs were calculated around R$ 10,989.00 per year per patient. When combining this cost to the information we have on the treatment of DM-originated wounds, it is stated that about R$ 59.4 billion/year is spent on it [3].

Scientific communities have been making multiple efforts in order to test new techniques and discoveries that aim at contributing to the improvement of this scenario. In 1996, the latex biomembrane (LBM) emerged as a promising element, because besides it being quite affordable, it also has physical and chemical characteristics that aid in healing with its neoangiogenic activity [9, 10].

LBM is a polyisoprene derivative, made of rubber latex (*Hevea brasiliensis*), a whitish secretion (also called “coagulated milky sap”) produced by the stem of the tree when it undergoes an incision in the bark, called “sangria.” This natural latex is composed of rubbery, nongummy hydrocarbon particles suspended in an aqueous serum phase, in which there is an occurrence of (on average) 36% hydrocarbons, 1.6% carbohydrates, 1.4% protein, 1% neutral lipids, 0.6% glycolipids plus phospholipids, 0.5% inorganic components, 58.5% water, and 0.4% other substances. Therefore, *Hevea brasiliensis* latex is a compound cytoplasmic system in which rubber particles and nonrubbery particles are dispersed in an aqueous cytosol phase [3, 11].

**Figure 1:**
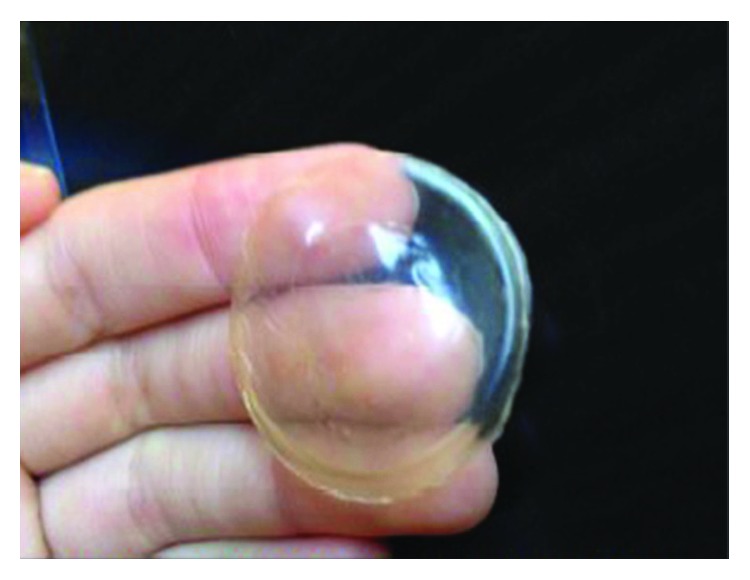
Latex biomembrane. This figure is reproduced from Ribeiro et al. [13], public domain.

Mrue et al. [12] emphasize that the great secret for LBM's angiogenesis (formation of new blood vessels) stimulation was only discovered because the polymer was not obtained through the traditional vulcanization method, which involves temperatures of 110°C to 125°C. In order to obtain the polymer, latex was collected with ammonia as the sole preservative, and centrifugation was then used to diminish its protein content, principally allergenic proteins. A sulfur composition was then added as the sole recovery agent; then, the latex was polymerized at low temperatures in a glass mold and then sterilized in ethylene oxide [3]. The result of this process is a biomembrane. The biomembrane is thin and elastic, has micropores that resemble human skin, and is easy to maneuver, as can be seen in Figure 1.

These characteristics help out in the wound repair process, particularly at the granulation tissue growth phase. This process can be divided into spatially and temporarily overlapping phases: (1) coagulation, (2) inflammation, (3) formation of granulation tissue (proliferative phase), and (4) remodeling or scar formation phase [14]. The endothelial cells are digested by molecules that make cell-cell and cell-matrix interactions. The endothelial cells are grouped and make a protrusion through the fragments of the basal membrane, at first forming solid rows of cells. Endothelial cells then begin to introduce cytoplasmic vacuoles, which are fused together at first among themselves and then with the neighboring cells, giving rise to new light. Factors by macrophages (factor of angiogenesis-derived macrophages), mast cells (heparin), platelets (platelet-derived growth factor, transforming growth factor beta), and fibroblasts (fibroblast growth factor) have all presented a positive effect on angiogenesis [14–16].

Latex, one of the materials used for the production of the therapeutic insoles features the advantages of its low market price, low pathogen transmission risk, neovascularization and tissue regeneration properties, and wide clinical-social applicability. Latex is extracted from the rubber tree *Hevea brasiliensis*, and it is both a healing substance and defense mechanism for this organism [17]. Numerous research which used natural latex as an implant on different tissues have presented satisfactory results, which motivate the development of new works in the area [13, 17–20]. Regarding this scope, personalized insoles made with natural latex have been used as an important tool for the reduction of plantar pressure in the treatment of patients with a diabetic foot [12, 17]. The research of Duff A. C. indicates that one in four of young diabetics (age 11-24) has increased plantar pressure and/or plantar blister (lump, tissue thickening—lat. plantar callus) [13, 20, 21]. The impacted areas are high-risk areas for development of some variety of foot conditions in adulthood. Biomechanical alterations increase the occurrence of development of fissures, blisters, and deformities. Limited range of motion in joints is commonly seen in diabetic patients [21]. Natural latex biomembrane (NLB) was proven to be an effective material in the reconstruction of the pericardium of dogs [22], in iatrogenic defects in the abdominal wall of rats [23], and in a neoangiogenic inductor in rabbit corneas [24, 25]. According to Balabanian et al. [26], granules of natural latex implanted inside the alveolar sockets of rats immediately after dental extraction demonstrated biocompatibility and become integrated with the alveolar bone, simultaneously accelerating bone formation undergoes and playing an important role in the healing process. Domingos et al. [27] have also demonstrated NLB's biocompatibility as a matrix for bladder augmentation in rabbits. The researchers affirm that it allows a progressive ingrowth of all layers of the bladder wall, raising epithelium and muscle regeneration without postoperative urinary leakage and with a slow rate of stone formation [25, 27].

Patients with chronic wounds come across multiple difficulties in this stage, such as vascular problems, atherosclerosis predisposition, renal insufficiency, and poor infection response [28, 29]. Diabetes mellitus (DM) is a set of metabolic alterations, whose main characteristic is the decompensation of glucose levels in the bloodstream, characterized as hyperglycemia or hypoglycemia [30]. There is a raised risk of infection due to the diabetic's inability to control the bacterial colonization site, and obviously, if the situation is not controlled, it will lead to a vicious circle, which will fatally evolve to the amputation stage. That being said, early intervention is essential in the wound-healing treatment's success [31, 32].

Candido ([33], p. 80) makes the following approach: antibiotics can produce toxic effects and inhibit healing and thus should be administered only when there is infection, and anti-inflammatories cause microcirculation vasoconstriction, reducing the inflammatory response and collagen synthesis, and should be used only when there is pain or inflammation. This analysis is important because otherwise there is a bigger probability of delay in the healing process and the rise of resistant bacteria.

It is at this healing stage that the LBM can contribute the most. From 1998 to 2000, this peptide- and angiogenesis-inducing material started being successfully used in some hospitals in the treatment of chronic wound patients suffering from diabetes. In some cases, the biomaterial is sprinkled on the wound, and a substance very similar to the VEGF (Vascular Endothelial Growth Factor) is released, rebuilding veins and arteries and carrying fuel so that the wound site can heal [12]. Therefore, this article intends to contribute to these studies through the gathering and exposition of scientific evidences on the successful use of LBM in the healing process, as well as its recommendations and prohibitions.

## 2. Materials and Methods

We conducted a study to review the scientific literature using the following databases: BIREME, LILACS, Burns, MEDLINE, PubMed, and SciELO. Publications from 2000 to 2016 were selected with the following keywords: “healing,” “diabetes mellitus,” “wounds,” and “latex biomembrane.” The inclusion criteria for the selection of publications were the publications in their entirety, being published and/or indexed in these databases in the period of 2000 to 2016, and that they addressed the issues of use of the rubber tree (*Hevea brasiliensis*) latex-derived biomembrane and being of free access. Articles published before 2000, articles that have not addressed the rubber tree (*Hevea brasiliensis*) latex-derived biomembrane, articles that presented DM-related issues that were not correlated to LBM, and those discussing issues related to healing and wounds without the use of the latex biomembrane were not considered for this study, thus being the exclusion criteria.

## 3. Results

Table 1 presents the results of the integrative review of the scientific literature. With the works selected through the inclusion and exclusion criteria, there were 18 publications found, among which were selected tests with 611 subjects that varied between humans, animals, and laboratory tests.

## 4. Discussion

Out of the 18 publications found, 17 papers presented satisfactory results with tests on 600 subjects (263 in humans, 1 laboratory test, and 336 in animals), i.e., 98% of cases with LBM implementation presented satisfactory results.

Sousa et al. [36], in 12 experiments with dogs, do not recommend the LBM for preperitoneal inguinoplasty due to encasement formation. However, the authors state that the biomembranes keep influencing the process of scarring with no fibrosis, no bruising, and no seroma and infection and with the induction of vascular neoformation and collagen deposition.

**Figure 2:**
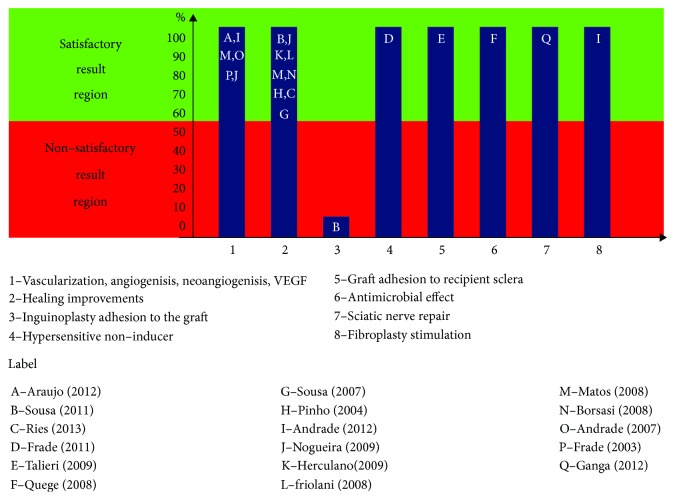
Main results obtained with the use of LBM in direct application, checked in the publication-surveyed 1 frame.

The publication results have been synthesized in Figure 2, featuring broadly the main contributions of the LBM.

It is observed that the latex biomembrane is highly recommended, because of the results acquired in the different publications. Six publications, with 128 human studies and 295 animal studies, refer to the LBM as a vascularization-, angiogenesis-, and VEGF-inducing agent, as well as eight publications (with 60 human studies, one laboratory test, and 129 animal studies) showing improvements in the healing process. Nevertheless, it still presents an antimicrobial effect, it encourages wound healing and the adherence to recipient sclerae, and it was proven to be as safe as a nonhypersensitivity-inducing bandage.

Frade [50] conducted clinical and immunohistopathological assessments in 21 casually selected patients, subject to the LBM application (14 patients), in a comparison with the classical treatment (7 patients), which consisted of an ointment with chloramphenicol and proteolytic enzymes (Fibrase) with the purpose of analyzing and comparing the histopathological and immunohistochemical alterations when it comes to different treatments.

In order to analyze the two scenarios, the biopsies were collected before and 30 days after Grochocki treatments. Biopsies were then split into two fragments: one for the immunohistopathological study and the other frozen at -70°C for immunohistochemical analysis. The results revealed that the use of the latex biomembrane facilitated the Grochocki care, and it was also proven to be an adequate alternative, due to its low cost and practicality of application. It was also observed that LBM induces a clinical and histopathological differentiation of healing points, with improved detection of growth factors such as VEGF and TGF1 (transforming growth factor 1).

**Figure 3:**
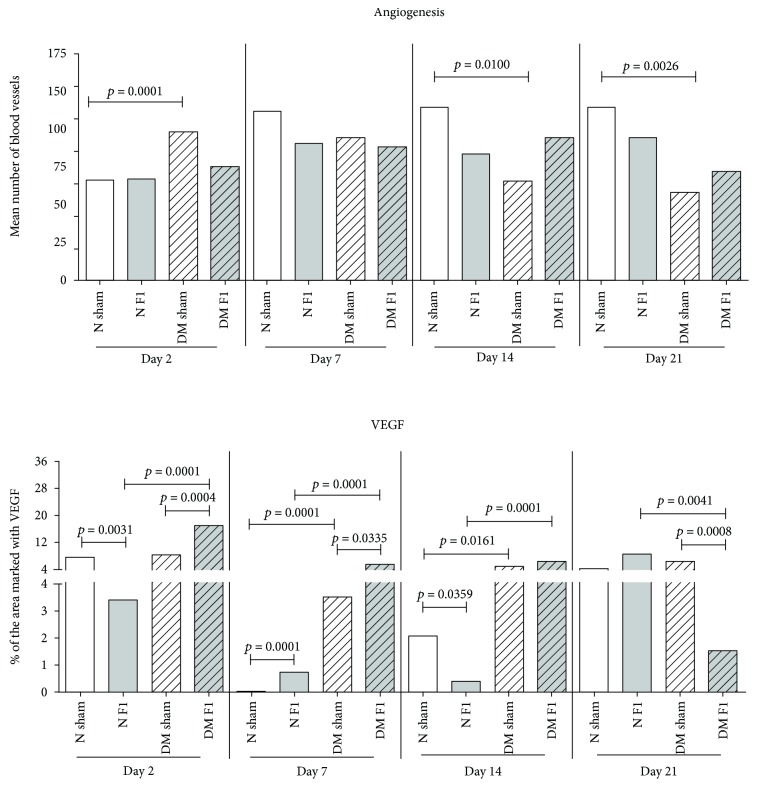
Characterization of angiogenesis increase and VEGF. This figure is adapted from Andrade [43], public domain.

A similar fact is evidenced in Andrade [43], where, in the assessing of skin ulcers due to diabetes mellitus (DM) in rats, it was found that the LBM performed as an important healing inducer where a latex stimulus to full reepithelialization was clinically observed. This stimulating inflammatory and oxidative stress phase favored the subsequent phases of wound healing, enhancing angiogenesis and VEGF in the 14th and 21st days, which certainly favored the reepithelialization. It is also assumed that there was a stimulus for fibroplasia in the 14th and 21st days and collagenase, as shown in Figure 3.

Thus, the essential factors which enabled the complete reepithelization of skin ulcers treated with F1 in diabetic rats, showing that the biomembrane (LBM) contributed favorably, were a larger recruitment of inflammatory cells, stimulation of the production of growth factors and cytokines, oxidative stress triggered until the 14th day, and the important fibroplasia and collagenase stimulus as well as the important signaling insulin activation, once reduced in diabetics.

In Frade et al. [37], there was an evaluation of the vegetable biomembrane's safety as a bandage in relation to latex hypersensitivity. Patients with cutaneous ulcers were selected, with the groups being control: low exposure to latex (sample = 17), high exposure to latex (sample = 14), and ulcerated using vegetable biomembrane (sample = 13) and experimental: ulcerated with no vegetable biomembrane (sample = 14) and new cases (sample = 9), all submitted for evaluation before and after 3 months using the biomembrane. All of them were submitted to clinical and epidemiological assessment regarding latex hypersensitivity and to a contact test (“patch test”). The study concluded that the vegetable biomembrane was safe to be used as a bandage, as it did not induce hypersensitivity reactions in the volunteers submitted to the “patch test.”

In Soares et al. [51], the treatment and healing of pressure ulcers using the latex biomembrane was studied, with substantial reduction of treatment time. Reis [42] presents the search for a new possibility for the healing of diabetic foot ulcers. In this sense, a then-unheard-of tissue neoformation-inducing system was developed for a diabetic foot, with a LED light circuit and using natural latex. This system involves a healing insole and an electronic tissue regeneration circuit. The insole's scarring effect is derived from the natural latex rubber tree *Hevea brasiliensis* and was individually custom made. This diabetic foot ulcer healing method is composed of the cooperative and simultaneous action of both biomaterial latex and the light irradiation of low-intensity LED lights.

Both features have properties and agents capable of inducing regeneration and tissue neoformation. When the patient is using the healing insole and the tissue regeneration electronic circuit, both of them will engage in the healing of the diabetic foot ulcer. This happens for two reasons: full contact of the ulcerated area with the latex and the low-intensity LED lights irradiating on the entire extent of the wound.

**Figure 4:**
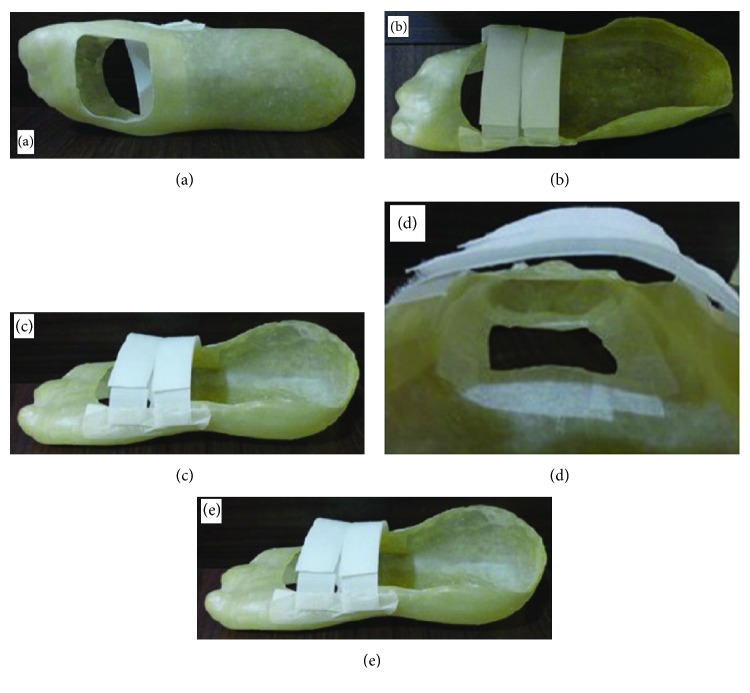
Healing insole: (a–c) various views of the sock with the gap; (d) microporous tape glued around the gap; (e) patient using the insole healing with tissue regeneration electronic circuit (circuit off). This figure is reproduced from Reis [42], public domain.

The insole's design was one of the important requirements throughout this project's preparation process. Since this is an insole that can be used either in hospitals or in everyday life, it is essential that it can provide the patient with the maximum possible comfort, softness, and well-being. Therefore, the making of this healing insole is personalized and individualized in its entirety, considering the anatomy and the specific features of the patient's foot such as size, shape, and proportion. This allows the LED light irradiation force cell (which is customized) to be installed in the exact wound spot in order to promote direct healing. In addition, customizing the insole makes it possible to perfectly accommodate the patient's feet possible deformities (foot dig or plan, bunions, claw-like fingers, and hammer, among others) if there is any, as shown in Figure 4.

Commercially sold insoles are made following just the numbering system and a standard model. They are not properly built for this, even if they may accommodate other foot health complications, and consequently, it is nearly impossible for them to be properly useful in the treatment of diabetic foot ulcers.

**Figure 5:**
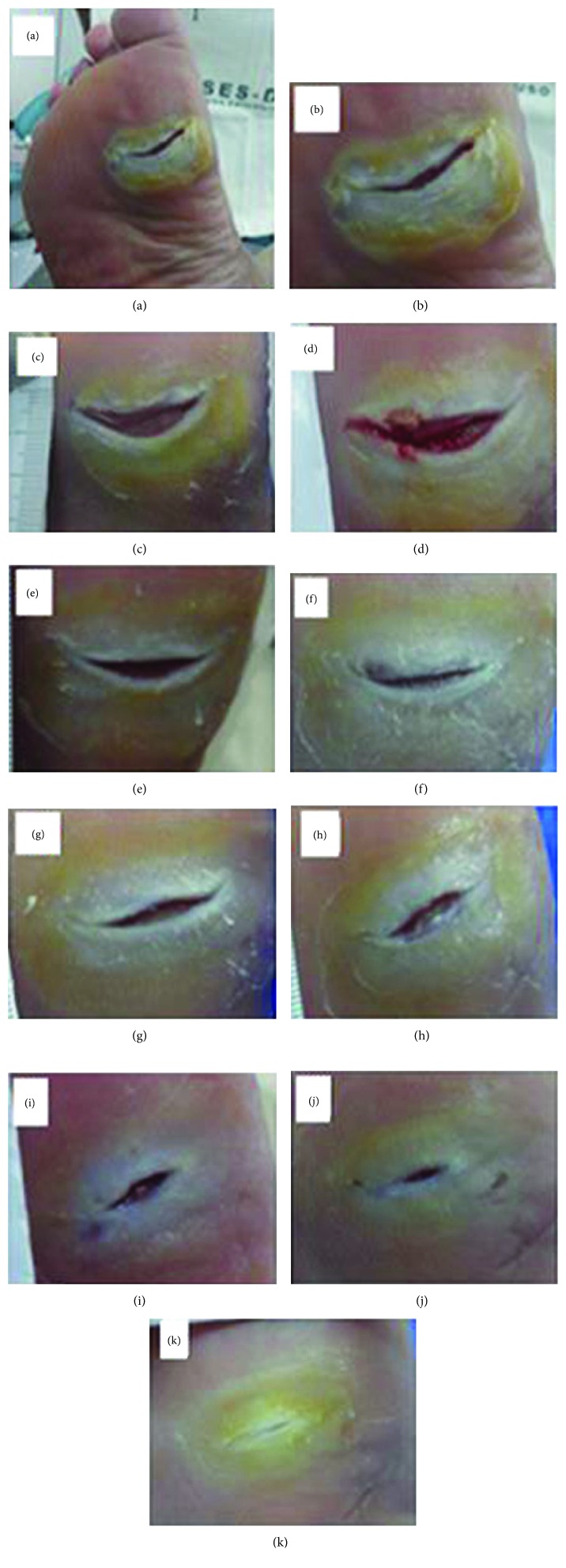
Photo clinical follow-up. Patient 1: control group: (a) ulcerated foot region; (b) pretreatment (initial); (c) posttreatment (1 week); (d) 2 weeks; (e) 3 weeks; (f) 4 weeks; (g) 5 weeks; (h) 6 weeks; (i) 7 weeks; (j) 8 weeks; (k) 9 weeks. This figure is reproduced from Reis [42], public domain.

**Figure 6:**
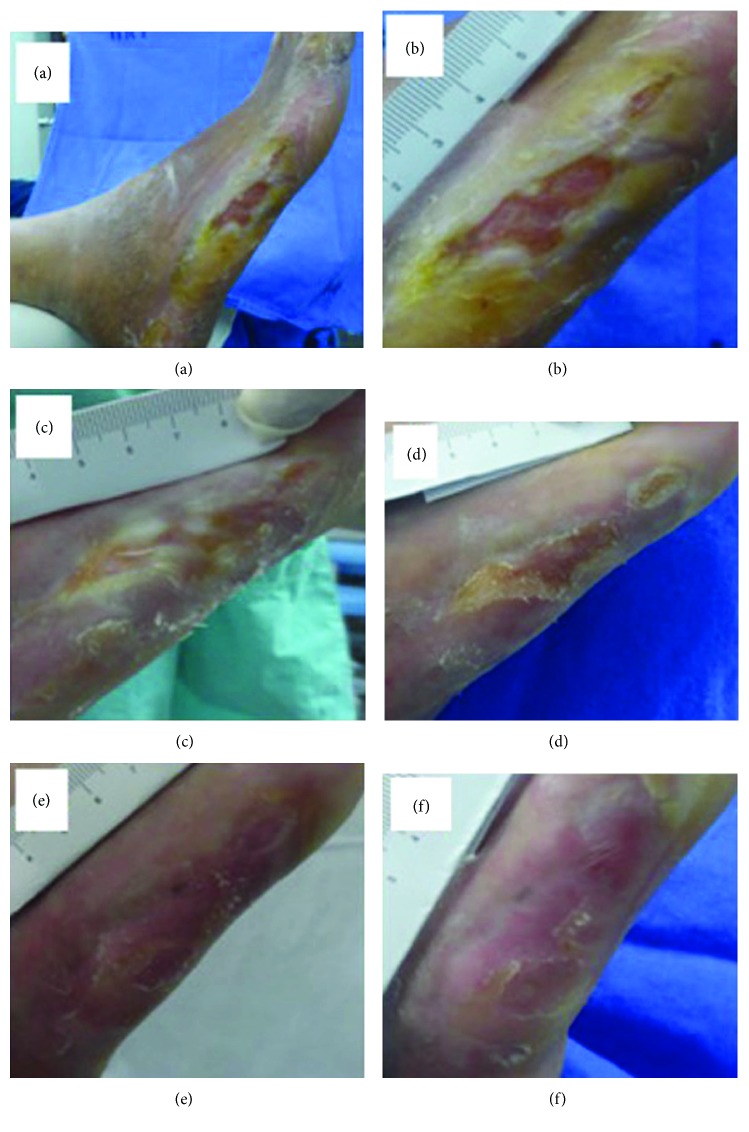
Photo clinical follow-up. Patient 2: experimental group: (a) ulcerated foot region; (b) early (before the tissue neoformation-inducing system); (c) posttreatment (after using the tissue neoformation-inducing system) (1 week); (d) 2 weeks; (e) 3 weeks; (f) 4 weeks. This figure is reproduced from Reis [42], public domain.

**Figure 7:**
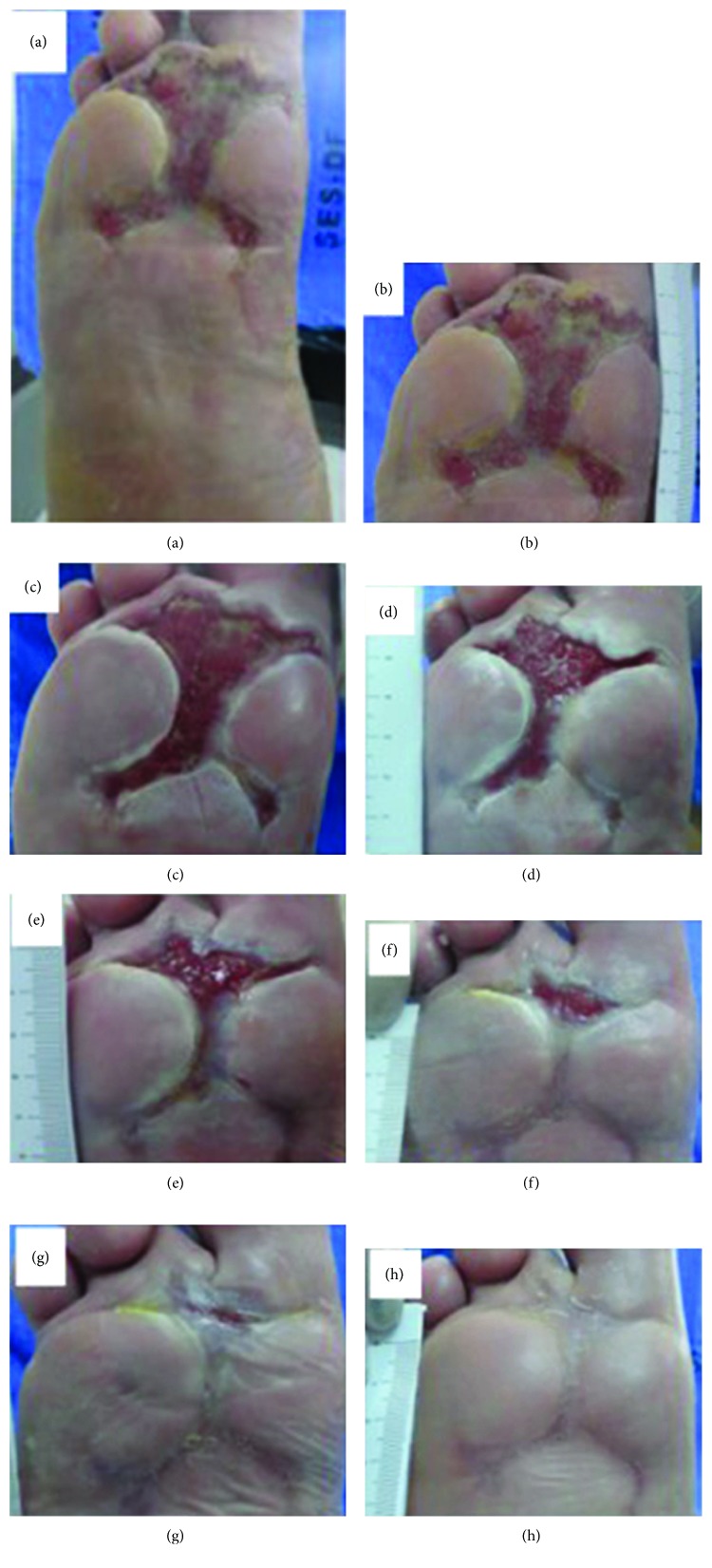
Photo clinical follow-up. Patient 3 (2 ulcer): experimental group: (a) ulcerated foot region; (b) early (before the tissue neoformation-inducing system); (c) posttreatment (after using the tissue neoformation-inducing system) (1 week); (d) 2 weeks; (e) 3 weeks; (f) 4 weeks; (g) 5 weeks; (h) 6 weeks. This figure is reproduced from Reis [42], public domain.

Another positive point of this invention is the low production cost, due to the material used in the making (biomaterial latex) and the fact that the tissue regeneration electronic circuit consists of LED lights. This invention has had satisfactory results, especially when it comes to reducing healing time, as demonstrated in Figures 5, 6, and 7.

**Figure 8:**
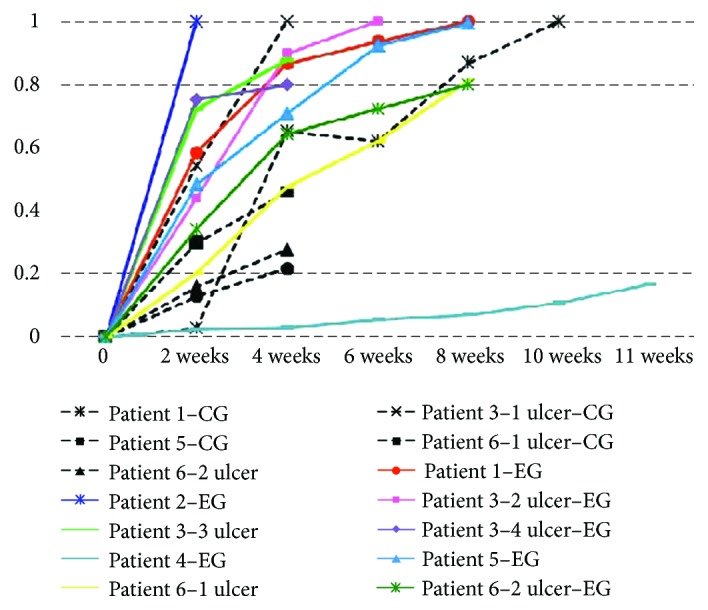
Evolution of the Ulcer Healing Rates (UHRs) in relation to the treatment time (in weeks) for the control group (CG) and the experimental group (EG). This figure is reproduced from Reis [42], public domain.

In Figure 8, the black dashes belong to the control group (CG), while the colored lines are the experimental group (EG). As it can be seen in this figure, in the second week of treatment, the EG's patient 2 had presented full reepithelization. Three patients (1 ulcer-CG; 2-EG ulcer), 1 (EG), and 1 (CG) also presented full reepithelization in the 4th, 6th, 8th, and 9th weeks. All patients showed healing evolution in all weeks, some with less and others with more intensity, except for patient 1 (CG), who in fact got worse between the 4th and 6th weeks. When comparing the two groups in the second week of treatment, it is possible to see that the best UHRs belonged to the 2 patients (EG), 1 (EG), 3 (CG and EG), and 5 (EG), while the other patients had UHRs below 0.4. It should be noted that patient 3's ulcer (1 ulcer-CG) was the smaller and the most superficial out of all ulcers in this study. When making the same comparison in week 4, it is possible to see that the highest UHRs belonged to patients 3 (CG and EG), 1 (EG), and 5 (EG). The worst result in all weeks was patient 4 (EG), whose chronic ulcer is harder to heal and has been there for the past 16 years. Despite all that, a small evolution in the healing process of their wounds was observed.

One of the reviews of this study was a comparison of the behavior of two different healing methods in the same patient. This refers to the patient 1, which was submitted to silver foam (CG) in their right foot ulcer (the metatarsals) and a neoformation tissue inducer system (EG) on their left foot ulcer (calcaneal region). Comparing the UHRs in both cases in the 2nd, 4th, 6th, and 8th weeks, patient 1 presented the best results in EG. This reveal that the tissue neoformation-inducing system favored an increase of healing better than foam with silver and in less time.

Costa et al. [8] made a survey on the cost of pressure ulcer (PU) treatment, which was calculated around R$ 915.75 per patient monthly and R$ 10,989.00 yearly in a hospital unit in Minas Gerais. The cost for the hospital is proportionally higher due to the number of patients that show up with PU and due to the possibility that the available resources might be used inappropriately. Considering the high investment in this treatment, it is important to properly control the materials needed in the care of PU; after all, the actions in these treatments should aim at cost reduction, the reduction of patient's suffering, and the possibility to provide them with humanized assistance.

The population with a higher risk of presenting pressure ulcer is, in most cases, people over 60 years old, because the skin becomes then much more sensitive due to the changes that come with the process of aging; white people, because “the black skin is more resistant to external damage”; the bedridden and/or restricted-to-wheelchair people; the malnourished; and those with very dry or very wet skin.

In Brazil, Biocure® is a registered trademark for the first national application of latex (Biomembrane® is manufactured by Pelenova Biotechnology S/A), which was founded in 2003. According to the directors of Pelenova, Freitas and Silva (2003), the Biocure® box with 20 units costs approximately U$ 30.00 (updated value, 2004 reference; R$ 28.50).

The similar products that come from the international market are much more expensive because they are based on the production of living cells, which involves very high industrial effort and expenses. In Japan, this medicine costs around U$ 500.00. Healing ointment gel with human hormone compounds (Becaplermin, Johnson & Johnson) is worth nearly U$ 350.00 and can only last for a few days of treatment. A human skin transplant patented by Novartis costs around U$ 1200.00, plus surgical expenses and the risk of it being rejected by the patient's body.

Therefore, it can be concluded that the cost for obtaining the LBM for tissue-healing treatment is considered low when compared with other traditional methods available on the market that are expensive and often end up in interrupted treatment, thus possibly worsening the patients' situations.

## 5. Final Considerations

By analyzing the experiments' results, it is possible to conclude that there are practical evidences of tissue healing with the use of the latex biomembrane, including in cases of chronic wounds from diabetes mellitus [39, 42, 43] and pressure ulcers [49, 50].

There were several cases that presented tissues' cellular reconstitution, revascularization, and function reestablishment after the burns, acting and reducing healing times in the inflammatory phase.

LBM's low production cost (and it is already being manufactured in Brazil by Pelenova Biotechnology S/A (Terenos/MS)) is another favorable aspect for this method to be made more popular. It is more affordable than traditional methods, and it also presents favorable results in a shorter period of time and in adverse conditions of treatments for healing.

The satisfying results make it possible to elucidate that there is still much to be studied and analyzed regarding the LBM besides the direct use in tissue healing processes. There is also the indirect use, with new applications, in order to take advantage of their biocompatible material properties, which demonstrated structural qualities (adaptability, elasticity, impermeability, and possibility of suture) and the absence of toxicity and allowed interaction between tissues (as demonstrated by the study on vascular prostheses [40]), whether as an inductor of sciatic nerve regeneration [35] or in bandages [37] for not inducing hypersensitivity and for having an antimicrobial effect [39].

All this considered, it is believed that the current results, as well as the forthcoming ones, will contribute considerably to the advancement of medicine, as well as in the treatment of many patients by giving them a faster healing time and an affordable cost that the majority of the population can afford.

## Figures and Tables

**Table 1:** Publication research.

Author	Study	Objective	Number of experiments			Results
Araujo et al. [34]	Anatomical and functional evaluation of tympanoplasty with the use of the transitional implant of the natural latex biomembrane from the rubber tree *Hevea brasiliensis*	This work aims at investigating the effects of the latex and silicone biomembrane in tympanic perforation restoration	107 humans			There was greater vascularity in the group with the transitional latex biomembrane implant. They presented good biocompatibility with the use of latex and silicone implants without affecting the rates of occurrence of infection, otorrhea, or otorrhagia. The proportion of tympanic membrane healing was equivalent in three groups, as well as the hearing improvement. Thus, the use of the implant caused a bigger graft vascularity process, with satisfactory interaction with human tympanic membrane tissues
Ganga et al. [35]	Sciatic nerve regeneration in rats through a conduit made from a natural latex membrane	To evaluate the NLB's capacity to accelerate and improve the quality of regeneration of a sciatic nerve cut in rats			40 Wistar rats	All morphological and functional analyses have shown that rats with the latex membrane recovered better than those with the autologous nerve: quality of printed shoeprints, treadmill performance, electrophysiological response, and histological quality of nerve regeneration. Thus, the data presented depicted behavioral and functional recovery in the rats that were implanted with the latex conduit through a complete morphological and physiological restoration of the sciatic nerve
Sousa et al. [36]	Morphological evaluation of the use of latex prosthesis in videolaparoscopic inguinoplasty: an experimental study in dogs	To evaluate (through videolaparoscopic inguinoplasty) the morphological aspects of the behavior of 4 types of latex biomembranes preperitoneally put in dogs			12 dogs	The biomembranes maintain the induction of the healing process fibrosis-free. They undergo encasement and, with the exception of the thin porous polyamide membrane, they are not incorporated in neighboring tissues. The latex biomembrane, alone, be it with or without polyamide, is not recommended for preperitoneal inguinoplasty
Frade et al. [37]	Vegetal biomembrane bandage and hypersensitivity	To evaluate bandage and hypersensitivity in the treatment of wounds with the latex membrane	67 humans			The biomembrane proved to be safe as bandaging, as it did not induce hypersensitivity
Talieri [38]	Natural latex graft in the healing of lamellar and penetrating sclerectomies in rabbits	This study aim at investigating the effects of natural latex with the 0.1% polylysine on the healing process of lamellar and penetrating sclerectomies in rabbits			24 rabbits	Great adhesion of the latex graft to the receptor's sclera
Quege et al. [39]	Comparison of the activity of essential fatty acids and the biomembrane on the microbiota of infected chronic wounds	To evaluate the highest efficiency between the latex membrane (Biocure) and AGE-based product (Dersani) in postleprosy treatment ulcers	8 humans			Dersani: positive antimicrobial effect in Enterobacter aerogenes Biocure: positive antimicrobial effect in *Pseudomonas aeruginosas*
Brandão et al. [40]	Latex-derived vascular prosthesis	To develop a new microperforated vascular prosthesis model, made of fabric covered with a natural rubber tree (*Hevea brasiliensis*) latex-derived compound, and to assess its patency rates, thrombogenicity, biocompatibility, and the process of healing, in addition to some mechanical properties (elasticity, adaptability, impermeability, and possibility of suture), using the expanded polytetrafluoroethylene prosthesis as a control in the same animal			15 dogs	The tissue and microperforated latex graft demonstrated structural qualities (adaptability, elasticity, impermeability, and possibility of suture) that were satisfying as a vascular substitute. It stimulated endothelial growth beyond the contact with the regions on the anastomosis and it was biocompatible with the dogs' arterial system, presenting appropriate tissue integration
Sousa et al. [41]	Latex biomembrane: new method for cavity flooring opened in tympanomastoidectomy	To study the performance of the biomembrane as an interface between the bone rim and the buffering material and to analyze its role in the epithelialization of the neocavity	54 humans			The use of the latex biomembrane proved to be an effective method in the neocavity coating, facilitating the removal of the cap and the epithelialization of the neocavity
Pinho et al. [24]	Experimental use of latex biomembrane in conjunctival reconstruction	To check the effect of the latex biomembrane in the conjunctival repair process			15 rabbits	As described in the literature for other tissues, the natural latex biomembrane also seems to favor the conjunctival scarring and neoangiogenesis. If these results repeat themselves in humans, the biomembrane could become a promising therapeutic feature in conjunctival reconstruction, particularly in cases where tissue revascularization is important
Reis [42]	Tissue neoformation-inducing system for diabetic feet with LED light circuit and use of natural latex	The goal is to evaluate the efficiency of the tissue neoformation-inducing system in the healing of diabetic foot ulcers. This system has been tested in patients with diabetic foot ulcer. Six patients with 11 ulcers were selected and then seen in the Diabetic Foot Center of HRT/DF. They constituted two distinct groups of treatment and study: control group and experimental group	6 humans			The clinical findings were analyzed qualitatively and quantitatively, demonstrating that the experimental group has higher results than the control group. Thus, tissue neoformation-inducing system may be considered an effective alternative for diabetic foot ulcer treatment, once it showed a high potential in healing induction
Andrade [43]	Tissue modifications and rubber latex *Hevea brasiliensis* F1 fraction action mechanisms in the healing of skin ulcers in diabetic rats	Diabetes (related to cellular stress) changes considerably the skin ulcer's healing process. The rubber tree *Hevea brasiliensis* latex has presented itself as especially relevant as an inducer of diabetes' compromised ulceration healing. It was clinically observed that the latex completely stimulates full reepithelialization. Tissue modifications were evaluated, as well as the latex protein fraction (F1) action mechanisms in the healing of skin ulcers in diabetic and nondiabetic rats. Initially, it was tested on the cytotoxicity of F1 in human fibroblast and keratinocyte cultures through the MTT colorimetric method			80 Wistar rats	Essential factors which enabled the reepithelization of the total skin ulcers treated with F1 in diabetic rats were a large recruitment of inflammatory cells, stimulation of the production of growth factors and cytokines, oxidative stress triggered until the 14th day, and the induction of collagenase and fibroplasia, as well as the significant activation of insulin signaling, once lowered in diabetics
Nogueira [44]	Oronasal fistula in dog: repair with a simple flap associated with a protein-purified angiogenic factor of hevea latex, aired with collagen sponge array—an experimental study	This experiment intended to use the purified protein fraction of hevea latex on the repair of inflicted oronasal communications, for experimental simulation of fistula, after the dogs' upper canines' dental extraction			6 dogs	The results were better quality healing, less inflammatory processes at the end of 21 days, less occurrence of suture dehiscence, and a greater amount of bone tissue in the alveoli, concluding that the use of the protein factor helps the repair process, making it faster and more efficient
Herculano [45]	Development of natural latex membranes for medical applications	In this work, we tested the latex biomembrane as an occlusive membrane for GBR with promising results		*In vitro* laboratory tests		The result indicated that the latex biomembrane could be used as an active membrane to fasten the healing process
Friolani [46]	The use of the latex hevea biomembrane (*Hevea brasiliensis*) in rabbits' diaphragmatic lesions: an experimental study	Considering the healing process accelerating properties the latex biomembrane has presented, this work aimed at evaluating the behavior of a natural latex biomembrane flap in diaphragmatic lesions experimentally induced in rabbits			15 rabbits	It was possible to conclude that the use of the latex membrane in repairing diaphragmatic lesions, due to its low cost and subsequent easiness to obtain and be used, not to mention its strength, presented a satisfactory answer in relation to the time of healing
Matos [47]	Effects of the natural latex biomembrane (*Hevea brasiliensis*) in Wistar rats submitted to body heat injury by scalding	Latex membrane biocompatibility			21 Wistar rats	The LBM (latex biomembrane) improved the healing in burned areas and stimulated neoangiogenesis, appearing then to be a promising therapeutic resource for healing of burned skin, in which tissue revascularization is important
Borsari [48]	Effects of the application of the natural latex biomembrane and frog skin extract (*Lithobates catesbiana*) (Shaw, 1802) in Wistar rats surgical wounds	A comparison between natural latex biomembrane's reaction whether isolated or with frog skin extract in cutaneous wounds. This work aims at evaluating the tissue reparation in the following aspects: biocompatibility, healing capacity, and possible complications			60 Wistar rats	All showed positive healing signs
Andrade [49]	Natural rubber tree *Hevea brasiliensis* latex biomembrane's activity in tissue neoformation in mice	The natural rubber tree *Hevea brasiliensis* latex biomembrane, used as a bandage in the treatment of chronic ulcers in humans, proves to be effective in debridement and to stimulate granulation and accelerating healing. Its mechanism of action is still unknown, making it important to evaluate its activity as an implant in tissue induction by comparing it to other implants and normal healing			60 C57BL/6 mice	It is concluded that the natural rubber tree *Hevea brasiliensis* latex biomembrane plays a significant role in the inflammatory phase of wound healing, thus being important in the neurophilic recruiting in the wound site, confirmed quantitatively by the concentration of myeloperoxidase and interleukin and immunohistochemistry. This fact seems to influence directly the subsequent phases of the healing process, confirmed by its ability to stimulate angiogenesis, which is probably not influenced by VEGF, and by stimulating fibroplasia TGF1 independent and with no modification on collagen production
Frade [50]	Foot ulcer: clinical characterization and immunohistopathological profile of healing in the presence of the natural rubber tree *Hevea brasiliensis* latex biomembrane	Foot ulcer is a very common disease in the elderly population. Numerous types of bandages are currently used for foot ulcer treatment with different indications, advantages and disadvantages, and which effectiveness is not well comprehended due to the discontinuity of the treatments and the costs involved in some situations. This work aims at evaluating the action of the latex biomembrane (LBM) in treating foot ulcers, which behaved like an efficient healing tissue inducer	21 humans			The global analysis of the data suggests that treatment with the biomembrane leads to scar tissue organization consequent to the increased production of cellular growth factors. Thus, the biomembrane is characterized as a good therapeutic option for foot ulcer due to the practicality of its application, low cost, and high potential in the induction of healing
